# Cystic fibrosis presenting as recurrent pancreatitis in a young child with a normal sweat test and pancreas divisum: a case report

**DOI:** 10.1186/1752-1947-2-176

**Published:** 2008-05-23

**Authors:** Laurie Conklin, Pamela L Zeitlin, Carmen Cuffari

**Affiliations:** 1 Division of Pediatric Gastroenterology and Nutrition, Johns Hopkins University School of Medicine, Baltimore, Maryland, USA; 2 Eudowood Division of Pediatric Respiratory Sciences, Johns Hopkins University School of Medicine, Baltimore, Maryland, USA

## Abstract

**Introduction:**

Pancreatitis is a rare manifestation of cystic fibrosis (CF) and may rarely be the presenting symptom in adolescent or adult patients with CF. We report a case of a 4 year-old female who initially presented with recurrent pancreatitis, a normal sweat test, and a diagnosis of pancreas divisum. She was subsequently diagnosed with cystic fibrosis at the age of 6 years, despite normal growth and no pulmonary symptoms, after nasal potential difference measurements suggested possible CF and two known CF-causing mutations (ΔF508 and L997F) were detected.

**Case Presentation:**

An otherwise healthy 4 year-old female developed chronic pancreatitis and was diagnosed with pancreas divisum. Sphincterotomy was performed without resolution of her pancreatitis. Sweat test was negative for cystic fibrosis, but measurement of nasal potential differences suggested possible cystic fibrosis. These results prompted extended Cystic Fibrosis Transmembrane Regulator Conductance (CFTR) mutational analysis that revealed a compound heterozygous mutation: ΔF508 and L997F.

**Conclusion:**

CFTR mutations should be considered in cases of chronic or recurrent pancreatitis despite a negative sweat test and the presence of pancreas divisum. Children with CFTR mutations may present with recurrent pancreatitis, lacking any other signs or symptoms of cystic fibrosis. It is possible that the combination of pancreas divisum and abnormal CFTR function may contribute to the severity and frequency of recurrent pancreatitis.

## Introduction

Pancreatitis is a rare manifestation of cystic fibrosis, affecting < 2% of patients with CF. Pancreatitis may be the presenting symptom in adolescent or adult patients with mild CF. We report a case of a 4 year-old female who initially presented with recurrent pancreatitis, pancreas divisum, and a normal sweat test. She was subsequently diagnosed with cystic fibrosis at the age of 6 years, despite normal growth and no pulmonary symptoms, after nasal potential difference measurements suggested possible CF and two known CF-causing mutations (ΔF508 and L997F) were detected (Table 1). In patients with CF pancreatitis, sweat chloride levels are often normal and even nasal potential differences may reveal only subtle abnormalities. Recurrent pancreatitis in children should raise suspicion for cystic fibrosis, despite a normal sweat test or a known diagnosis of pancreas divisum.

**Table 1:** Nasal potential difference measurements at basal, change after amiloride, chloride, and isoproteronol infusions. Results were suggestive of possible cystic fibrosis.

	NORMAL	CF	Pt's Left Nare	Pt's Right Nare
**Basal (mV)**	18.2 ± 8.3	-45 ± 11	-21	-35
**Δ Amiloride**	10.5 ± 6	29.8 ± 11	9	33
**Δ Low Chloride**	-14.1 ± 9	2.2 ± 2.3	-2	-12
**Δ Isoproteronol**	-9.6 ± 5	0.9 ± 2.2	-6	-2

## Case presentation

A 4 year-old female presented with a two-month history of recurrent episodes of pancreatitis. Her clinical symptoms were diffuse, episodic abdominal pain in the absence of vomiting, weight loss, fevers or joint pain. Stools were described as bulky and green. On physical examination of the abdomen, there was mild tenderness in the epigastrium, but no peritoneal signs nor hepatosplenomegaly. At the time of initial presentation, the patient's serum lipase and amylase were >30,000 U/L and >18,000 U/L, respectively. The remainder of her laboratory workup was normal, including serum cholesterol and triglycerides. An abdominal ultrasound showed a hyperechoic pancreas consistent with pancreatitis, and a hepatobiliary system that appeared normal. The patient received supportive care, including bowel rest and hydration, and was ultimately discharged on a low fat diet once her serum amylase and lipase normalized. Her previous investigations included a normal sweat chloride test and a normal endoscopic retrograde cholangiopancreatography (ERCP).

The patient was subsequently readmitted one month later with another episode of clinical pancreatitis. On examination of her previous ERCP images, the possibility of short ventral duct was suspected and a repeat ERCP demonstrated pancreatic duct divisum. She underwent minor papilla sphincterotomy and pancreatic duct stent placement. Despite the sphincterotomy, she was again readmitted to hospital on three separate occasions over 12 months with clinical and biochemical pancreatitis, prompting the institution of pancreatic enzyme supplementation with meals and snacks.

**Figure 1:**
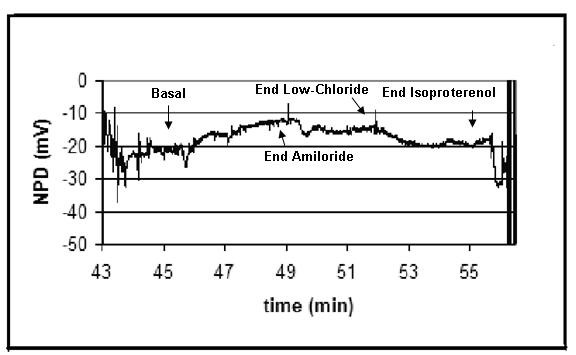
**Nasal potential difference measurements, patient's left nare**. Patients with cystic fibrosis can be differentiated from controls by the measurement of nasal potential differences (NPD). In this study, nasal potential differences were measured using a standardized operating procedure that was developed by a Cystic Fibrosis Foundation clinical trials network to be followed by all sites that perform collaborative studies [1].

Cystic fibrosis (CF) was suspected, but sweat chloride was 23 mEq/L and a CF gene mutation analysis was positive for only one copy of the ΔF508 mutation. She then underwent nasal potential difference measurements. Mean baseline potential difference was -28 mV (range -21 to -35 mV). Superfusion of amiloride to lower the sodium potential blocked 55% of the potential difference, and was within normal limits. The low chloride and isoproterenol responses were equivocal, but suggestive of CF, thereby raising concerns for an unidentified CF mutation on the second allele. (Figure 1, Chart 1) [1].

Extended Cystic Fibrosis Transmembrane Receptor (CFTR) mutation analysis showed that the patient was positive for the L997F mutation in addition to the ΔF508, both known cystic fibrosis-causing mutations. She had no respiratory complaints and had repeated pulmonary function testing that was normal. Interestingly, her father is of Irish ancestry and her mother is of Maltese descent. Both were shown to carry a CF mutation, the mother carrying the more obscure L997F mutation. Three of four siblings were later found to carry one or the other mutation and remain healthy.

## Discussion

Cystic fibrosis (CF) is a common inherited and clinically heterogenous multisystemic disorder that affects glands and secretory epithelia. Although most patients have chronic lung disease and pancreatic insufficiency, 20% of patients are able to digest and absorb fat normally, and as a consequence are at risk for recurrent pancreatitis. In this unique patient population, pancreatitis is frequently the clinical presentation that leads to the diagnosis of CF. These patients are typically diagnosed with CF later in childhood, as the cases are clinically less severe and usually lack sinopulmonary disease.

Pancreatitis is a rare manifestation of cystic fibrosis, affecting < 2% of patients with CF. The incidence of symptomatic pancreatitis in patients with cystic fibrosis has been shown to be only 1–2%. A minority of patients with CF (13–15%) express the pancreatic sufficient phenotype. These patients are known to carry a greater risk of pancreatitis and have genotypes causing less severe loss of function. Over 1000 CFTR mutations have been identified and have been archived on an online database. [2] About half of patients with CF in the United States are ΔF508 homozygotes, and another 40% are compound heterozygotes, who have one ΔF508 allele plus one less common CF- allele. Mutations in Class 1, 2 (ΔF508), or 3 are severe and associated with pancreatic insufficiency. Compound heterozygotes with a mild Class 4 or 5 mutations, unlike ΔF508 homozygotes, typically have pancreatic sufficiency and are frequently diagnosed at an older age due to the milder phenotypic presentation and lack of suspicion for the diagnosis. Multiple cystic fibrosis gene mutations are associated with chronic pancreatitis, including the rare L997F mutation found in our patient [3,4].

The L997F (missense substitution of leucine with phenylalanine at position 997) is a highly conserved residue in transmembrane domain 9. Both heterozygosity for L997F and compound heterozygosity for other CFTR mutations have been associated with idiopathic disseminated bronchiectasis, recurrent pancreatitis, and hypertrypsinemia in infants. L997F was identified in 4 (12.5%) out of 32 patients with idiopathic pancreatitis, and in 4 (8%) of 49 infants with hypertrypsinemia. Among the 4 patients with recurrent pancreatitis, just one was a compound heterozygote (L997F/ΔF508). The others included one L997F/5T, and two with L997F/no mutation. In this same study, among the mothers of these children with CF who had recurrent pancreatitis or hypertrypsinemia, most were found to be carriers of ΔF 508 gene mutations. Interestingly, none of the mothers carried the L997F mutation [5,6]. According to the CF Consensus Statement from 1998, these studies would support categorizing L997F as a "CF-causing mutation" associated with the increased probability of acquiring pancreatic ductular obstruction and an increased risk for recurrent pancreatitis, despite normal sweat chloride testing [7].

In another study of 14 adults diagnosed with idiopathic chronic pancreatitis or recurrent acute pancreatitis, the L997F mutation was identified in 3 patients. [4] On the other hand, one report disputed the association, describing a case of homozygosity for L997F in a child with a normal clinical phenotype, normal sweat test, and normal intestinal chloride transport [8]. A recent case report identified a 5 year-old Pakistani child with cystic fibrosis and high sweat chloride levels who was found to have the L997F mutation. That patient had symptoms compatible with CF without a history of chronic recurrent pancreatitis [9].

In our patient, the availability of nasal transepithelial potential difference measurements ultimately led to the clinical suspicion of compound heterozygosity for the CF mutation. Respiratory epithelium, including nasal epithelium, regulates the transport of ions such as sodium (Na^+^) and chloride (Cl^-^). Because of the abnormal Cl^- ^transport in CF, nasal potential difference (PD) measurements in these patients reveal a different pattern than in patients without the disease. Three features distinguish CF from controls: 1) higher basal PD, reflecting enhanced Na+ transport secondary to relative Cl^- ^impermeability 2) greater inhibition of PD after nasal perfusion with amiloride, a Na^+ ^channel inhibitor, and 3) little or no change in change of Cl^- ^transport in response to perfusion with a Cl^-^-free solution along with isoproteronol, (which reflects an absence of CFTR-mediated Cl^- ^secretion). Particularly, the chloride secretory response to chloride free and isoproteronol has high sensitivity and specificity for normal *versus *CF or atypical CF. [10] This method has increased the ability to detect patients with compound heterozygous CF, which can present more atypically.

Patients with compound heterozygous CF gene mutations, unlike ΔF508 homozygotes, typically have pancreatic sufficiency and are frequently diagnosed at an older age due to the absence of overt pulmonary symptomatology. In these patients, sweat chloride levels are often normal and nasal potential differences may also show only subtle perturbation in normal CFTR function, as was demonstrated in our patient. As commercial genetic testing for extended CFTR mutation analysis is now available, it is important to suspect a CFTR mutation in a patient with idiopathic chronic pancreatitis.

Interestingly, our patient was diagnosed with pancreas divisum prior to being diagnosed with cystic fibrosis. It is known that pancreas divisum is found in the healthy general population with a prevalence of 5–10%[11]. Why certain patients with pancreas divisum develop pancreatitis and not others is not known. One study has suggested a link between CFTR dysfunction and recurrent acute pancreatitis in patients with pancreas divisum. In this study, among those patients with recurrent pancreatitis and pancreatic divisum, the nasal evoked potentials were intermediate between those healthy patients and those with overt CF [12]. In another study comparing incidence of CFTR mutations between patients with pancreatitis and controls undergoing ERCP, 22% of patients with pancreatitis and pancreas divisium had a CFTR mutation compared with 0% of controls with pancreas divisum [13]. The combination of pancreatic divisum and abnormal CFTR function may contribute to the severity and frequency of recurrent pancreatitis. The treatment of recurrent pancreatitis with sphincterotomy and stent placement is controversial. The benefit of minor papillotomy and stent placement in patients with pancreatic divisum is controversial with a high rate for necessary reintervention. In a retrospective review of the largest experience reported to date of endoscopic therapy for pancreas divisum at a referral center, two types of sphincterotomy procedures were compared, needle-knife sphincterotomy (NKS) and the standard pull-type sphincterotomy (PTS). The reintervention rate was similar for both at 29 and 26% respectively. It is not clear whether the need for reintervention was secondary to a high rate of stenosis or if the pancreas divisum was not the underlying cause for the pancreatitis [14].

Our patient had no response to sphincterotomy. The combination of dietary management and pancreatic enzyme supplementation reduced the frequency of pancreatitis paroxysms. Unfortunately, our patient shows radiological signs of chronic pancreatitis, despite therapy. All of these measures were implemented to improve our patient's quality of life. Furthermore, this child's diagnosis ultimately led to genetic testing of her 4 siblings that ultimately led to the identification of 3 CF gene mutation carriers.

## Conclusion

Chronic or recurrent pancreatitis in children should raise suspicion for CFTR mutations, despite a normal sweat test or the presence of an existing diagnosis of pancreas divisum. Since CFTR mutation analysis has become commercially available, we anticipate that the number of idiopathic pancreatitis patients in pediatrics should decrease. Among patients with pancreatic divisum refractory to sphincterotomy, identification of the coexistence of CFTR dysfunction may facilitate a more practical therapeutic approach, including dietary modification, pancreatic enzyme supplementation and close clinical follow-up.

## Competing interests

The authors declare that they have no competing interests.

## Authors' contributions

LC drafted the manuscript, CC and PZ participated in the care of the patient and interpretation of the testing. All authors read and approved the final manuscript.

## Consent

Written informed consent was obtained from the parent of the patient for publication of this case report and accompanying images. A copy of the written consent is available for review by the Editor-in-Chief of this journal.
